# Implementation of a molecular tumour board in LATAM: the impact on treatment decisions for patients evaluated at Instituto Alexander Fleming, Argentina

**DOI:** 10.3332/ecancer.2021.1312

**Published:** 2021-11-01

**Authors:** Martín Osvaldo Angel, Carmen Pupareli, Tomas Soule, Florencia Tsou, Mariano Leiva, Federico Losco, Federico Esteso, Juan Manuel O´Connor, Romina Luca, Fernando Petracci, Romina Girotti, Yamil Damián Mahmoud, Claudio Martín, Matías Chacón

**Affiliations:** 1 Genitourinary Oncology Unit, Instituto Alexander Fleming, Cramer 1180, Ciudad Autonoma de Buenos Aires, C1426ANZ, Argentina; 2 Thoracic Oncology Unit, Instituto Alexander Fleming, Cramer 1180, Ciudad Autonoma de Buenos Aires, C1426ANZ, Argentina; 3 Sarcoma and Melanoma Oncology Unit, Instituto Alexander Fleming, Cramer 1180, Ciudad Autonoma de Buenos Aires, C1426ANZ, Argentina; 4 Head and Neck Oncology Unit, Instituto Alexander Fleming, Cramer 1180, Ciudad Autonoma de Buenos Aires, C1426ANZ, Argentina; 5 Gastrointestinal Oncology Unit, Instituto Alexander Fleming, Cramer 1180, Ciudad Autonoma de Buenos Aires, C1426ANZ, Argentina; 6 Breast Cancer Unit, Instituto Alexander Fleming, Cramer 1180, Ciudad Autonoma de Buenos Aires, C1426ANZ, Argentina; 7 Laboratorio de Inmuno-Oncología Traslacional, Instituto de Biología y Medicina Experimental (IBYME), Vuelta de Obligado 2490, Ciudad Autonoma de Buenos Aires, C1428ADN, Argentina; 8 Clinical Oncology, Instituto Alexander Fleming, Cramer 1180, Ciudad Autonoma de Buenos Aires, C1426ANZ, Argentina; a https://orcid.org/0000-0002-1463-8887; b https://orcid.org/0000-0002-0322-0434; c https://orcid.org/0000-0001-5084-3012; d https://orcid.org/0000-0003-1977-9846; e https://orcid.org/0000-0002-6975-5466; f https://orcid.org/0000-0002-7701-3331; g https://orcid.org/0000-0001-7254-5892; h https://orcid.org/0000-0003-4135-7332; i https://orcid.org/0000-0001-6872-4185

**Keywords:** molecular tumour board, precision medicine, molecular profile

## Abstract

**Background:**

The role of the molecular tumour board (MTB) is to recommend personalised therapy for patients with cancer beyond standard-of-care treatment. A comprehensive molecular analysis of the tumour in a molecular pathology laboratory is important for all targeted therapies approaches. Here we report the 1-year experience of the Instituto Alexander Fleming Molecular Tumour Board.

**Patients and methods:**

The MTB of the Instituto Alexander Fleming was launched in December 2019 in a monthly meeting. In each interactive monthly session, five cases were presented and discussed by the members. These cases were referred by the treating oncologists. The MTB recommendations were sent to each physician individually, and to the rest of the meeting participants. This was discussed with the patients/families by the treating oncologist. The final decision to choose therapy was left to the treating physicians. Of the 32 patients presented at MTB, 28 (87.5%) had potentially actionable alterations and only 4 (12.5%) had no actionable mutation. Six (19%) patients received a local regulatory agency approved drug recommendation, nine (28%) patients received an off-label approval treatment recommendation and three (9%) patients did not receive the treatment due to access and reimbursement of the drug.

**Conclusion:**

In most of the cases evaluated, the MTB was able to provide treatment recommendations based on targetable genetic alterations. Molecular-guided extended personalised patient care is effective for a small but clinically significant proportion of patients in challenging clinical situations. We believe that the implementation of a MTB is feasible in the Latin America (LATAM) region.

## Background

The identification of molecular targets has allowed the personalisation of medicine. The first example arises from the discovery of the c-Kit activation pathway in gastrointestinal (GI) tumours and the use of imatinib for its treatment [1]. Only one patient was enough to demonstrate this proof of concept; this is, from our point of view, the birth of precision medicine. The use of targeted therapies in oncology is gaining ground, and oncologists are striving every day to implement them to change the paradigm of oncology practice that requires large-scale clinical trials to change therapeutic behaviours [2].

Later, with advances in the understanding of tumour biology and the discovery of oncogenic activation pathways, the oncologist began to implement biomarker-based treatment strategy. The landmark milestone of genomics in oncology was The Cancer Genome Atlas project that was able to molecularly characterise over 20,000 tumours [3]. Different tumour models have followed this molecular characterisation strategy and multiple therapeutic targets have been identified [4]. Some examples of malignant melanoma arise with the identification of activating mutations in the BRAF oncogene, being one of the most frequent genetic alterations in melanoma (50%). BRAF mutations result in the constitutive activation of the BRAF kinase and, consequently, the subsequent activation of the mitogen-activated protein kinase signalling pathway that regulates proliferation, growth and cell differentiation [5, 6]. Treatment with BRAF and MEK inhibitors has increased overall survival in melanoma.

Lung cancer is another good example for molecular subtyping. The epidermal growth factor receptor (EGFR) represents an important signalling pathway that regulates tumorigenesis and cell survival and is frequently overexpressed in the development and progression of non-small cell lung cancer (NSCLC) [7, 8]. This was followed by the identification of other activation pathways such as ALK, ROS, MET, BRAF and RET alterations [9–12]. Multiple targeted therapies have been approved for these molecular alterations.

With the landmark approvals of pembrolizumab for the treatment of patients whose tumours have high microsatellite instability, larotrectinib and entrectinib for those harbouring neurotrophic tyrosine receptor kinase (NTRK) fusions, a regulatory pathway has been created to facilitate the approval of histology-agnostic indications [13, 14].

The molecular similarities between histology and anatomical location of cancer types provide basis for developing strategies for future therapeutic research. This approach can only be carried out by a multidisciplinary team with a comprehensive molecular vision: the molecular tumour board (MTB). The role of the MTB is to recommend personalised therapy for patients with cancer beyond standard-of-care treatment [15]. A comprehensive molecular analysis of the tumour in a molecular pathology laboratory is important for all targeted therapies approaches. However, the interpretation of the molecular results is crucial and potential therapeutic conclusions can only be drawn by considering the clinical situation and within a setting of oncological experience. MTBs are becoming more common worldwide.

We established an MTB to interpret individual patient’s tumour genetic profiles and provide treatment recommendations. Here we report the 1-year experience of the Instituto Alexander Fleming Molecular Tumour Board. We are a private oncological institute in Buenos Aires, Argentina, specialised in solid and haematological tumours with a higher volume of patients per year.

## Patients and methods

**Figure 1.**
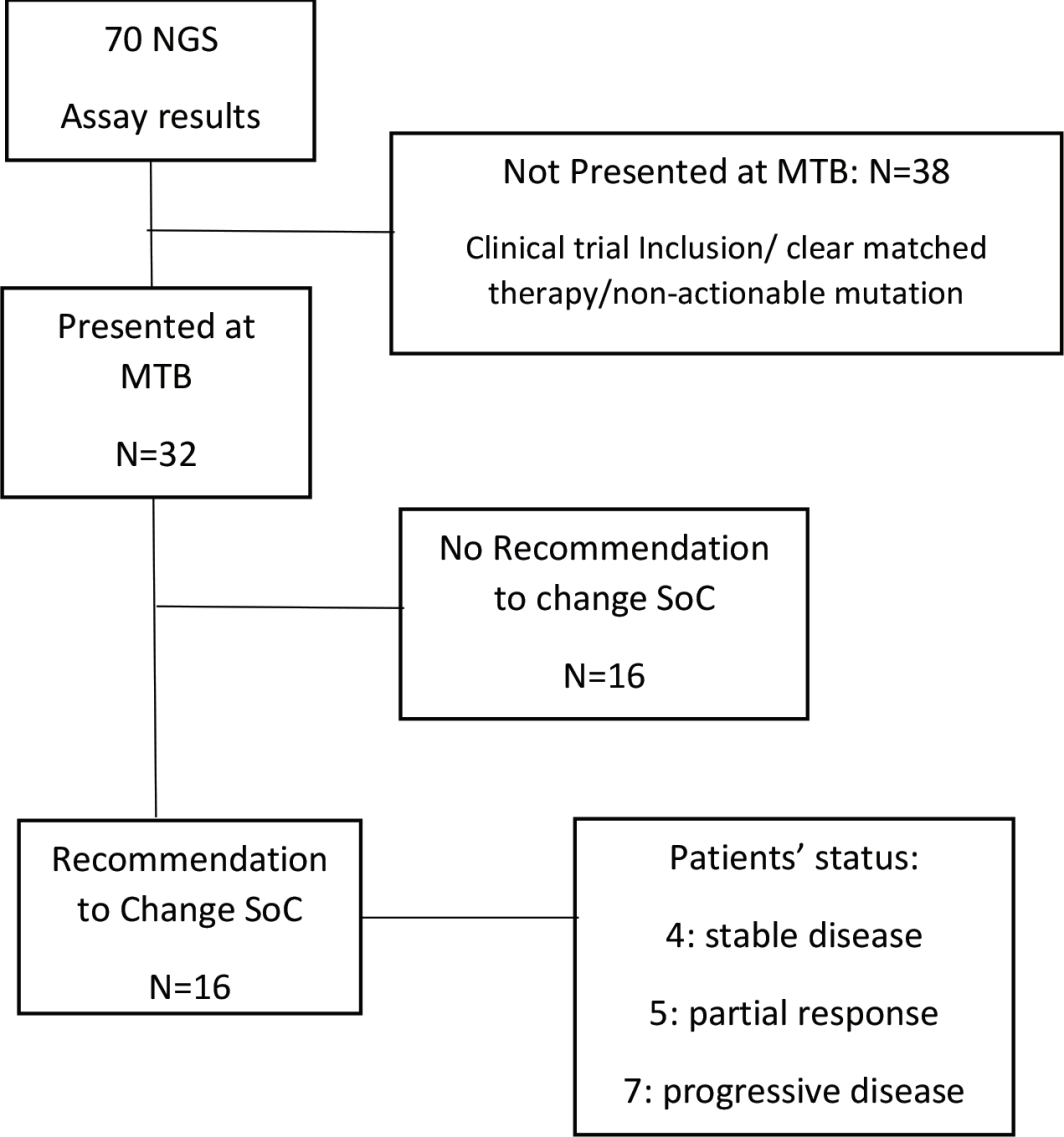
Workflow of the MTB at Instituto Alexander Fleming (period: December 2019 through December 2020). SoC, Standard of care; NGS, Next generation sequencing; MTB, Molecular tumour board.

The MTB of the Instituto Alexander Fleming called IAF_Molecular was launched in December 2019 in a monthly meeting. The MTB involves medical oncologists, pathologist, clinical trials investigators, genetic counsellors, molecular biologists and bioinformatics professionals. In each interactive monthly session, five cases were presented and discussed by the members. These cases were referred by the treating oncologists. All information was de-identified according to the local ethics committee. Patients were informed about the MTB decision-making process when their case was referred for discussion. Patient’s information required: primary tumour type, stage at diagnosis, previous lines of treatment, performance status, molecular and/or immune-histochemical biomarkers, etc. This was followed by a discussion of the results of the molecular profile and the implications for each case by the molecular biologist. The information discussed included the alterations detected in each sample, their level of characterisation and potential actionability. Targeted therapies or immunotherapies that matched each detected and approved alteration in the patient’s tumour type or in another tumour type were also analysed, as well as the open enrolment of clinical trials with genomic compatibility. This was only a consultative discussion. The MTB recommendations were sent to each physician individually, and to the rest of the meeting participants, by email and were kept on the shared drive for future reference. This was discussed with the patients/families by the treating oncologist. The final decision to choose therapy was left to the treating physicians (Figure 1).

Regarding the comprehensive genomic profiling (CGP) tests, all patients presented at MTB had performed a commercially available test as FoundationOne®CDx, (Liquid or Heme) or Caris®.

## Results

### Patient characteristics

Patients whose cases were selected for CGP had a range of different solid tumour types (n = 70): NSCLC (n = 13), breast cancer (n = 4), GI cancer (n = 34), genitourinary cancer (n = 3), unknown primary (n = 3), sarcomas (n = 3), head and neck cancer (n = 6) and gynaecological cancer (n = 4). Patients who were candidates for any of the open clinical trials at our site, or non-actionable mutation detected, and with clear matches to Food and Drug Administration as well as national regulatory agency (Administración Nacional de Medicamentos, Alimentos y Tecnología Médica) approved therapies in their tumour type were excluded for MTB discussion. Cases were selected for presentation to the MTB where the specific genomic mutation was not a direct match to an approved treatment or available clinical trial. Patients included for MTB discussion (n = 32). Amongst patients presented, the majority were NSCLC (28%, 9/32) followed by breast cancer (12.5%, 4/32), colorectal cancer (12.5%, 4/32), pancreatic adenocarcinoma (12.5%, 4/32), carcinoma of unknown primary (CUP) (9%, 3/32) and others tumour types (25%, 8/32) including oesophageal carcinoma, sarcomas, mesothelioma, cholangiocarcinoma, kidney and prostate cancer. Demographics of patients discussed are represented in Table 1. Median age was 57.8 years (interquartile range (IQR): 52–68.7) and 1:1 male to female ratio.

Median number of previous treatments lines was 2 (IQR: 2–3). All patients presented an Eastern Cooperative Oncology Group (ECOG) performance score of 0 at the time of MTB presentation.

### Genomic alterations and potential treatment options identified

**Figure 2.**
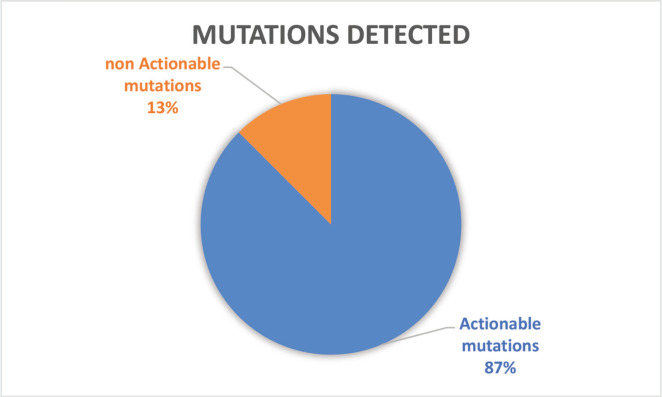
Number of patients with actionable mutations presented at MTB.

**Figure 3.**
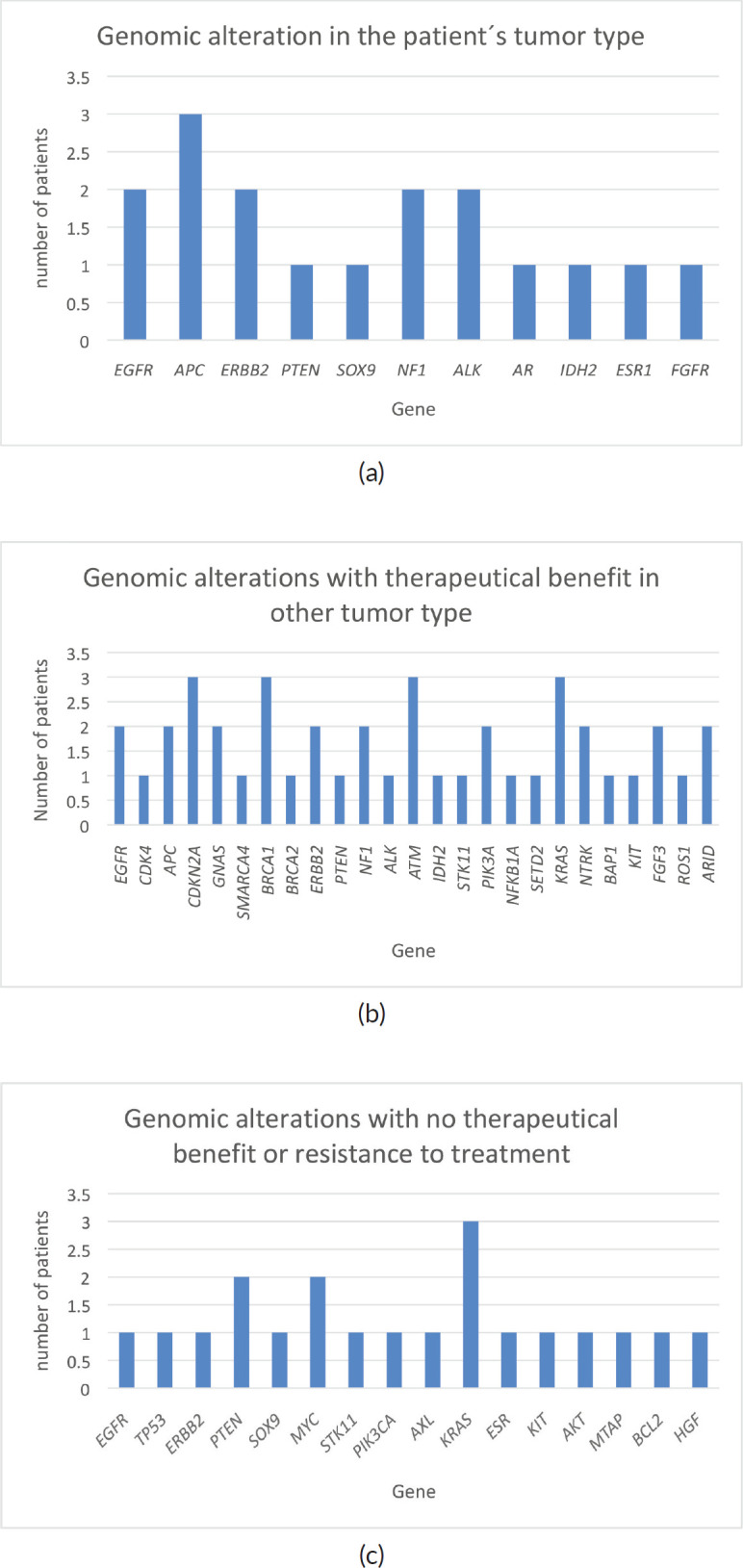
Genomics alterations detected in patients presented at MTB. (a): Genomic alteration with potential benefit in patient’s tumour type. (b): Genomic alterations with potential therapeutical benefit in other tumour types. (c): Genomic alterations with no therapeutic benefit or resistance to treatment.

**Figure 4.**
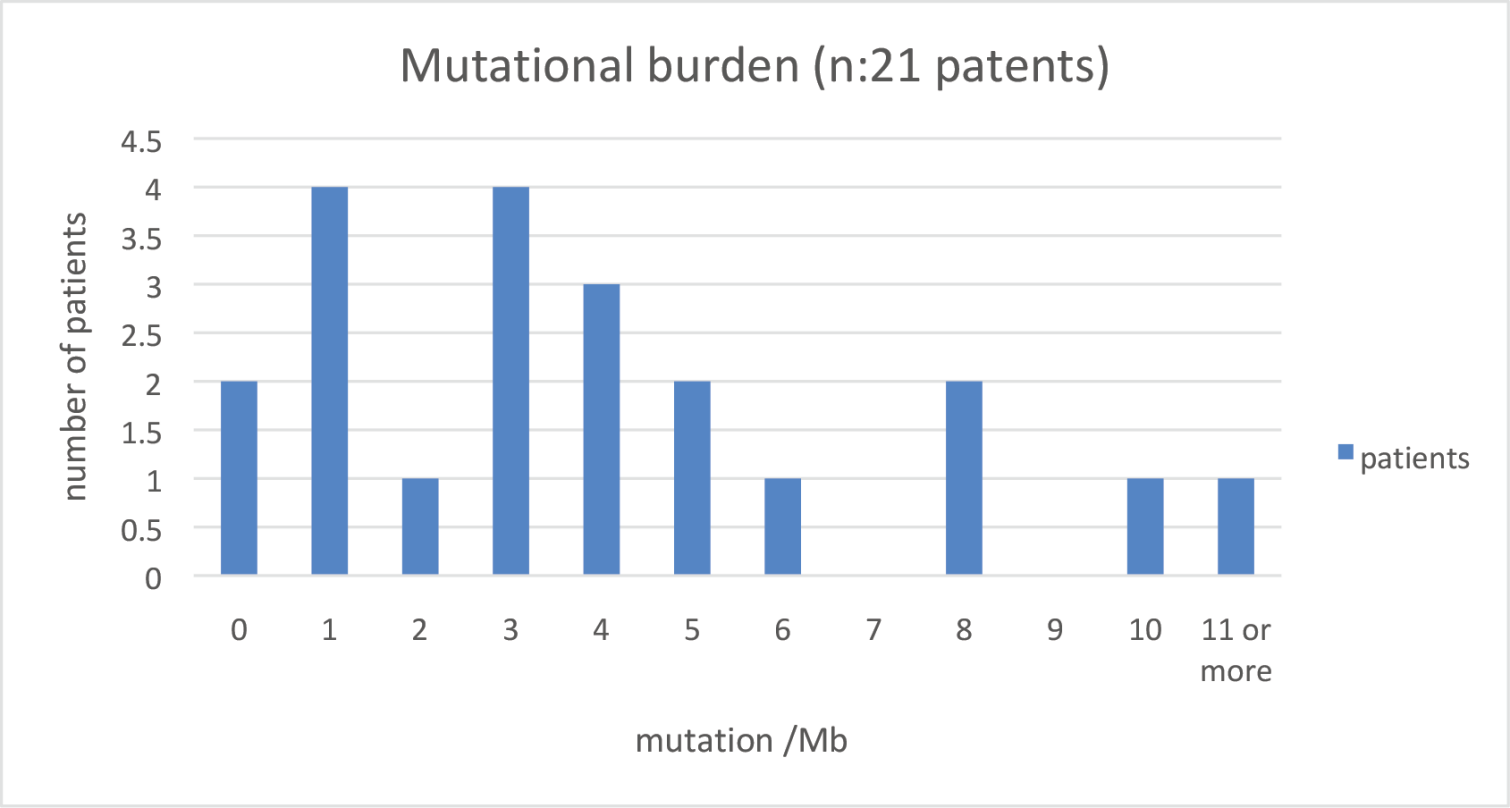
Number of patients according to mutations/Mb.

Of the 32 patients presented at MTB, 28 patients (87.5%) had potentially actionable alterations and only 4 (12.5%) had no actionable mutation (Figure 2). Fifteen cases (46.8%) had alterations with therapeutic benefit in their tumour type and 17 (53.1%) had alterations with matched therapy in another tumour type. In eight patients (25%), more than one potentially actionable alterations were identified. Twenty cases (62.5%) had an identified resistance alteration (Figure 3). All the patients presented at MTB had microsatellite status stable or undetermined. The median number of mutations per megabase determined as tumour mutational burden (TMB) was 4.3 mut/mb (IQR: 1.0–5.65) (Figure 4). Only two patients have high TMB.

### Treatment assignments and patient outcomes

Treatment recommendations in MTB are detailed in Table 1. Six (19%) patients received a local regulatory agency approved drug recommendation, nine (28%) patients received an off-label approval treatment recommendation and three (9%) patients did not receive the treatment due to access and reimbursement of the drug. One patient received an un-approved drug under an early access programme.

Amongst patients presented at the MTB, and those in which treatment recommendation was adopted, the median progression free survival was 15.25 months. According to best response obtained, median PFS in patients achieving disease control (SD + PR) was 16.4 months (95% confidence interval: 1.1 -19.4 months, p = 0.04).

## Discussion

We believe that the implementation of a MTB committee in our region is feasible and also necessary. The region comprises almost 8.5% of the world’s population. During the last decades, Latin America (LATAM) has experienced an increase in life expectancy and the subsequent ageing of the population. This change inevitably results in a higher incidence of cancer [16, 17]. Regarding molecular profile of different tumours, although there is a clear country and continental variability in terms of frequency, this difference is not significant and the overall incidence of different targets mutations in LATAM does not differ from the rest of the world. (EGFR and ALK in NSCLC [18], BRCA1 and BRCA2 in breast and ovarian cancer [19] and homologous recombinant deficiency in prostate cancer [20].) The request for genomic profiling is increasingly frequent worldwide [21], also in our institution, mainly as a diagnostic tool, so clinical oncologists need the MTB space for a better understanding of the report and to identify the oncogenic pathway that is dominating in the patient. Amongst the advantages that can be identified by having an MTB, is to have a common space for discussion of clinical cases, and to share the scientific evidence available in a tumour model to be applied in another type of tumour with an identified mutation. This cross communication between different branches of oncology allows us to optimise treatment and guide the best therapy to our patients.

The presence of activating mutations of certain signalling pathways are the ones that dominate the progression of the disease; therefore, the acquisition of new knowledge of signalling pathways and the action of these pathways to determine the treatment as well as to identify drug resistance mutations are also useful. In this sense, the incorporation of molecular biologists and bioinformaticians to our MTB also helped us with this point. Amongst the patients presented at the MTB, at least 16 known resistance mutations were identified, so the treatment recommendation issued by the committee was to continue with standard treatment despite the availability of an actionable mutation.

Another point of interest of our MTB was the incorporation of a genetic counsellor in oncology, which helped us to identify mutations that potentially present a germline implication and require follow-up of the patient and family members [22]. Amongst those present at MTB, at least five patients were identified, who were referred for germline testing, mainly when mutations in the BRCA and ATM genes were identified.

The strongest point of our committee that we can identify as very helpful is the ability to collect individual patient data and perform an aggregate analysis according to the identified mutations. This allowed us to standardise therapeutic behaviours in certain types of tumours, as well as to be able to design strategies for the treatment of certain types of cancer.

Differences in MTB interpretation and recommendation vary by centre, access to the clinical trial and other factors [23–25]. To date, there are no validated workflows to provide recommendations for the identified molecular targets. The strength of our committee is the ability to collect data from individual patients and perform an aggregate analysis according to the identified mutations. This allowed us to standardise therapeutic behaviours in certain tumours types, as well as to be able to design strategies for the treatment of certain types of cancer.

## Conclusion

In most of the cases evaluated, MTB was able to provide treatment recommendations based on actionable genetic alterations. Although the number of patients presented in our report is still small, our observations suggest that MTBs bring together relevant expertise and are crucial to patient care.

## Conflicts of interest

None of the authors have conflicts of interest to declare.

## Funding

The authors have not declared a specific grant for this research from any funding agency in the public, commercial or not-for-profit sectors.

## Figures and Tables

**Table 1.** Clinical and genomic characteristics of patients’ treatment based on MTB discussion.

Patient	Diagnosis	Genomic alteration	MTB recommendation	PFS (months)	Best response
1	NSCLC	EGFR K745-E746ins	Afatinib	29.25	SD
2	NSCLC	GNAS p.Q227L	Trametinib	10.75	PD
3	CRC	ERBB2	Trastuzumab/pertuzumab	1.58	PD
4	NSCLC	ALK	Alectinib	38.08	PR
5	Pancreatic adenocarcinoma	ATM	Olaparib	9.67	PR
6	Urothelial	STK11	Everolimus	1.55	PD
7	CRC	BRCA1	Olaparib	Never done	PR
8	Ductal carcinoma (breast)	ERBB2	Trastuzumab Pertuzumab	13.58	PD
9	CUP	KIT	Avapritinib	17.00	PD
10	NSLC	EGFR	Osimertinib	37.75	PR
11	mCRPC	AR amplification	Abiraterone	Never done	PR
12	NSCLC	ALK fusion	Lorlatinib	23.83	PD
13	CUP	ERBB2	Trastuzumab/pertuzumab	15.25	PD
14	Ductal carcinoma (breast)	FGF19 amplification	Infigratinib	Never done	SD
15	Bone sarcoma	FGFR1 amplification	Pazopanib	7.42	SD
16	Cholangiocarcinoma	IDH1	Ivosidenib	Never done	SD
Median PFS: 15.25 months					

NSCLC, Non-small cell lung carcinoma; CRC, Colorectal carcinoma; CUP, Carcinoma of unknown primary; mCRPC, Metastatic castration resistant prostate cancer; SD, Stable disease; PD, Progressive disease; PR, Partial response; PFS, Progression-free survival; AR, androgen receptor.
